# Prevalence of Congenital Anomalies of the Upper Limbs in Brazil: a descriptive cross-sectional study

**DOI:** 10.1590/1516-3180.2023.0349.R1.08042024

**Published:** 2024-06-17

**Authors:** Samuel Ricardo Batista Moura, Luis Renato Nakachima, João Baptista Gomes dos Santos, João Carlos Belloti, Carlos Henrique Fernandes, Flavio Faloppa, Vinicius Ynoe de Moraes, Rodrigo Guerra Sabongi

**Affiliations:** I Hand Surgeon, Department of Orthopedics and Traumatology, Escola Paulista de Medicina - Universidade Federal de São Paulo (EPM-UNIFESP), São Paulo (SP), Brazil; II Adjunct Professor, Department of Orthopedics and Traumatology. Discipline of Hand and Upper Limb Surgery, Escola Paulista de Medicina - Universidade Federal de São Paulo (EPMUNIFESP), São Paulo (SP), Brazil; III Adjunct Professor, Department of Orthopedics and Traumatology, Escola Paulista de Medicina - Universidade Federal de São Paulo (EPMUNIFESP), São Paulo (SP), Brazil; IV Adjunct Professor, Department of Orthopedics and Traumatology, Escola Paulista de Medicina - Universidade Federal de São Paulo (EPMUNIFESP), São Paulo (SP), Brazil.; V Adjunct Professor, Department of Orthopedics and Traumatology, Escola Paulista de Medicina - Universidade Federal de São Paulo (EPMUNIFESP), São Paulo (SP), Brazil.; VI Full Professor, Department of Orthopedics and Traumatology, Escola Paulista de Medicina - Universidade Federal de São Paulo (EPMUNIFESP), São Paulo (SP), Brazil; VII Orthopedic Surgeon, Department of Orthopedics and Traumatology, Escola Paulista de Medicina - Universidade Federal de São Paulo (EPMUNIFESP), São Paulo (SP), Brazil.; VIII Hand Surgeon, Department of Orthopedics and Traumatology, Escola Paulista de Medicina - Universidade Federal de São Paulo (EPM-UNIFESP), São Paulo (SP), Brazil.

**Keywords:** Epidemiology, Upper extremity, Congenital abnormalities, Hand deformities, Upper Limb, Prevalence, Congenital upper limb anomaly

## Abstract

**BACKGROUND:**

Congenital Anomalies of the Upper Limb (CAUL) are a group of structural or functional abnormalities that develop during intrauterine life and can lead to limb dysfunction.

**OBJECTIVES:**

To analyze the prevalence of congenital anomalies of the upper limbs in Brazil and assess maternal and neonatal variables.

**DESIGN AND SETTING:**

A cross-sectional, descriptive study was conducted on congenital upper limb malformations among live births across Brazil.

**METHODS:**

The study spanned from 2010 to 2019. Data were sourced from the Department of Informatics of the Unified Health System (DATASUS) and the Live Birth Information System (SINASC) portal. Analyses focused on the information reported in field 41 of the Live Birth Declaration Form entered into the computerized system.

**RESULTS:**

The most common anomaly in Brazil was supernumerary fingers, classified as ICD-Q69.0, affecting 11,708 children, with a prevalence of 4.02 per 10,000 live births. Mothers aged over 40 years had a 36% higher prevalence of having children with CAUL than mothers under 40 years old (OR = 1.36; 95% CI 1.19-1.56). Newborns weighing ≥ 2,499 g were 2.64 times more likely to have CAUL compared to those weighing ≥ 2,500 g (OR = 2.64; 95% CI 2.55-2.73).

**CONCLUSION:**

There was an observed increase in the reporting of CAUL cases over the decade studied. This trend serves as an alert for health agencies, as understanding the prevalence of CAUL and its associated factors is crucial for preventive medicine.

## INTRODUCTION

Congenital anomalies (CAs) are structural or functional alterations in embryos or fetuses that result from factors occurring before birth.^1^ These developmental changes can be genetic, environmental, unknown, or multifactorial in origin.^2^ CAs affect 1% to 3% of newborns, with approximately 10% of these cases involving congenital anomalies of the upper limb (CAUL).^3,4^

CAUL varies from minor isolated alterations with minimal impact on limb function to significant changes affecting vital organs. Monitoring these anomalies can help reduce morbidity and mortality in affected patients.^5^

Prevalence studies are essential in epidemiology for planning preventive public health measures. Currently, there are no studies on the prevalence of congenital upper limb anomalies in Brazil. This study aims to fill that gap using data from a Brazilian database. Worldwide, several databases monitor these anomalies, including the “Latin American Collaborative Study of Congenital Malformations,” a universal database in Latin America that supports clinical and epidemiological research.^6^

In Brazil, the Department of Informatics of the Unified Health System (DATASUS) under the Ministry of Health developed the “Live Birth Information System” (SINASC) in 1990 to collect epidemiological data on births nationwide. The standard document utilized is the “Live Birth Certificate” (DNV), which is mandatory for all live births regardless of delivery circumstances.^7^

Understanding the causes of CAs, especially those that are preventable, is crucial. Specific strategies in health policies can elucidate the increase in the proportion of deaths caused by CAs. The chronic nature of CAUL incurs significant socioeconomic costs and necessitates long-term multidisciplinary care. Increased investment in support strategies for patients with CAs is necessary, and further studies are needed to identify primary causes and associated factors.^2^

Epidemiological data on CAUL are vital for the development, planning, and monitoring of public health strategies. Studies on etiology and prevention depend on high-quality epidemiological data.^8^ The accuracy of an epidemiological study hinges on understanding the studied population and the authenticity of the collected data.^9^ This study hypothesizes that the national prevalence data for CAUL are consistent with those collected globally.

## OBJECTIVE

The objective of this study was to analyze the prevalence of CAUL in Brazil from 2010 to 2019, utilizing the DATASUS database, and to evaluate the associated maternal and neonatal variables.

## METHODS

### Research design

This descriptive cross-sectional study examined cases of CAUL in newborns in Brazil from 2010 to 2019, adhering to the Strengthening the Reporting of Observational Studies in Epidemiology (STROBE) guidelines.

### Data-gathering period

Data were collected from September to October 2021 and extracted from the Department of Informatics of the Unified Health System - DATASUS (available at http://www2.datasus.gov.br).^10^ This database compiles information from the mandatory Live Birth Certificate (DNV) for all live births in Brazil, maintained in the SINASC system.

### Selection criteria

The study variables included demographic details, types of upper limb congenital anomalies, and maternal and newborn variables as recorded in DATASUS. The data from the live birth certificates, which contain 41 fields divided into seven blocks, were utilized. Field 41 specifies congenital anomalies as noted by the delivery personnel or a neonatologist. Following Chapter XVII, titled “Congenital Malformations, Deformities, and Chromosomal Abnormalit” all anomalies were recorded non-hierarchically, with a detailed description of the codes from the International Classification of Diseases (ICD-10).

### Data-gathering

The Swanson classification was employed to categorize CAUL,^11^ grouping similar deficiency patterns based on specific embryological faults. The categories included: (I) failure of formation, (II) failure of differentiation, (III) duplication, (IV) overgrowth, (V) undergrowth, (VI) congenital constriction band syndrome, and (VII) generalized skeletal abnormalities. Anomalies were grouped according to their corresponding ICD-10 codes.

All ICD-10 codes corresponding to CAUL diagnosed at birth were selected. Diagnoses were grouped to categorize anomalies according to related pathologies (Table 1).

**Table 1:** ICD-10 grouping in relation to the type of CAUL Brazil, 2010-2019

* CAUL	** ICD-10
	Q71.0 - Complete congenital absence of the upper limb(s)
	Q71.1 - Congenital absence of the arm and forearm, with hand presente
	Q71.2 - Congenital absence of the forearm and hand
	Q71.3- Congenital absence of the hand and finger(s)
	Q71.4 - Radius longitudinal reduction defect Club hand (congenital) / Radial hand
**Training Deficiency**	
	Q71.5 - Longitudinal reduction defect of the ulna [ulna]
	Q71.6 - Lobster claw hand
	Q71.8 - Other upper limb reduction defects Congenital shortening of the upper limb(s)
	Q71.9 Defect due to reduction of the upper limb, unspecified
	Q70.0- Coalescence of the fingers (fused fingers)/Complex syndactyly of the fingers with synostosis
**Differentiation Deficiency**	Q70.1 - Webbed fingers / Simple syndactyly of the fingers without synostosis
	Q70.4- Polysyndactyly
**Duplication Deficiency**	Q69.0- Supernumerary finger(s)
	Q69.1 - Supernumerary thumb(s)
	Q68.1 - Other congenital musculoskeletal deformities - Congenital hand deformity
**Widespread anomalies**	Q74.0 - Other congenital malformations of the upper limb(s), including the shoulder girdle
	Q74.3 - Multiple arthrogryposis congenita

The variables of interest selected for analysis pertained to the period and place of birth, maternal data (age, education, gestational duration, type of delivery, type of pregnancy, and prenatal visits), and newborn variables (Apgar scores at 1 and 5 min, sex, birth weight, and race/ethnicity).

### Data processing and analysis

Data were collected between February and June 2022. Based on these data, the total prevalence of CAUL in DATASUS from 2010 to 2019, as well as the specific prevalence according to maternal and newborn variables, were calculated using the following formula:


$$\frac{\text{Number of malformed live births in the period}2010-2019\;\times \;10.000}{\text{Number of live births in the in the period}2010-2019}$$

Data were extracted, organized, and encoded in a spreadsheet using Microsoft Excel, version 16.0, developed by Microsoft (Redmond, Washington, United States), and then processed using the Statistical Package for the Social Sciences (SPSS) software, version 23.0, developed by International Business Machines Corporation (IBM) (New York, United States).

Due to the limitations of individualized data, a linear trend model was applied. The three-point moving average (MM (3)) smoothing method was utilized to enhance the visualization of the linear trend. Simple univariate linear regression analysis was conducted for predictive modeling and to estimate future values. No discernible patterns of cyclic or irregular components were identified during the subjective analysis of the graph; therefore, no cyclical analysis was performed. As the source data were annual, it was impossible to identify a seasonal component throughout the year. Each year of occurrence was used as the independent variable, and the ratio of live births with upper limb malformations to the total number of live births per year was used as the dependent variable. An overall analysis was conducted for Brazil, and a regional analysis was performed for the country (North, South, East, and West). For statistical inference, a statistically significant difference was considered at a type I error rate of P < 0.05.

This study was submitted to and approved by the Research Ethics Committee of the Federal University of São Paulo (UNIFESP) with the approval number 5.036.478.

## RESULTS

Between 2010 and 2019, Brazil registered 29,157,184 live births, of which 238,571 had general CAs. The Southeast region recorded the highest number of live births, while the Central-West had the fewest during this period.

In total, 216,801 congenital anomalies were identified, including 21,770 cases of CAUL. The anomalies were categorized into four types based on related pathologies: formation defects, with 3,938 cases (18%); differentiation, with the fewest cases at 1,572 (7.2%); duplication, encompassing the majority with 12,012 cases (55.0%); and generalized anomalies, with 4,248 cases (19.5%).

The number of newborns with CAUL was analyzed separately by ICD codes and country. The national prevalence of CAUL was 7.5 per 10,000 LBs. The most prevalent anomaly was supernumerary fingers, represented by ICD-Q69.0, affecting 11,708 children (a prevalence of 4.02 per 10,000 live births). In contrast, the anomaly with the lowest national prevalence was the longitudinal reduction defect of the ulna, represented by ICD-Q71.5, with a prevalence of 0.01 per 10,000 live births (Table 2).

**Table 2:** Prevalence of congenital malformations of the upper limbs by regions of Brazil for every 10 thousand LB (2010-2019)

ICD – CAUL	Regiões do Brasil						
	North	Northeast	West Central	Southeast	South	Total	
	LB*						
	3.127.884	8.286.407	2.371.666	11.482.289	3.888.938	29.157.184	
	CAUL (**)	CAUL (**)	CAUL (**)	CAUL (**)	CAUL (**)	CAUL (**)	Prevalence
Q69.0- Supernumerary finger(s);	441	3.435	731	6.137	964	11.708	4,02
Q68.1 - Congenital hand deformity;	215	682	212	1.104	443	2.656	0,91
Q71.3 - Congenital absence of the hand and finger(s);	147	392	169	724	280	1.712	0,59
Q74.0- Other Congenital Malformations of the Upper Limbs	136	283	72	583	149	1.223	0,42
Q71.8- Other upper limb reduction defects. Congenital shortening of the upper limb(s);	67	157	51	331	110	716	0,25
Q70.4- Polysyndactyly	44	143	40	271	109	607	0,21
Q70.0- Coalescence of the fingers - Complex syndactyly of the fingers with synostosis	43	152	51	245	99	590	0,20
Q719-Defect of upper limb reduction, unspecified.	55	123	37	246	75	536	0,18
Q71.2- Congenital absence of the forearm and hand	31	92	30	159	72	384	0,13
Q70.1- Webbed fingers Simple syndactyly of the fingers without synostosis	24	124	39	146	42	375	0,13
074.3- Multiple Congenital Arthrogryposis	20	123	15	161	50	369	0,13
Q69.1- Supernumerary thumb(s)	20	59	32	150	43	304	0,10
Q71.6- Lobster claw hand	28	63	16	91	30	228	0,08
Q71.0- Complete congenital absence of the upper limb(s)	19	47	20	67	22	175	0,06
Q71.4- Radius longitudinal reduction defect | Club hand (congenital) | Radial hand	09	18	06	50	10	93	0,03
Q71.1- Congenital absence of the arm and forearm, with hand present	05	16	08	24	07	60	0,02
Q71.5- Longitudinal reduction defect of the ulna [ulna];	02	06	04	18	04	34	0,01
**Total**	1.306	5.915	1.533	10.507	2.509	21.770	7,5

(*) LB= live births;

(**) CAUL=congenital anomaly of the upper limbs

Regional prevalence of CAUL per 10,000 LBs by ICD-10 code from 2010 to 2019, showed the Southeast having the highest rate of 9.15. The Northeast had the second-highest prevalence (Table 2).

The anomaly of supernumerary fingers (ICD-Q69.0) had the highest regional prevalence in the Southeast, at 5.34 per 10,000 LBs. The congenital hand deformity (ICD-Q68.1), the second most prevalent anomaly nationwide, had its highest prevalence in the Southern region, at 1.14 per 10,000 live births (Table 3).

**Table 3:** Prevalence de CAUL by region (per 10,000 LB) by ICD-10 code between 2010 and 2019

CAUL*	N	NE	WC	SE	S
Q69.0- Supernumerary finger(s);	1,41	4,15	3,08	5,34	2,48
Q68.1 - Congenital hand deformity;	0,69	0,82	0,89	0,96	1,14
Q71.3 - Congenital absence of the hand and finger(s);	0,47	0,47	0,71	0,63	0,72
Q74.0- Other Congenital Malformations of the Upper Limbs	0,43	0,34	0,30	0,51	0,38
Q71.8- Other upper limb reduction defects. Congenital shortening of the upper limb(s);	0,21	0,19	0,22	0,29	0,28
Q70.4- Polysyndactyly	0,14	0,17	0,17	0,24	0,28
Q70.0- Coalescence of the fingers - Complex syndactyly of the fingers with synostosis	0,14	0,18	0,22	0,21	0,25
Q719-Defect of upper limb reduction, unspecified.	0,18	0,15	0,16	0,21	0,19
Q71.2- Congenital absence of the forearm and hand	0,10	0,11	0,13	0,14	0,19
Q70.1- Webbed fingers Simple syndactyly of the fingers without synostosis	0,08	0,15	0,16	0,13	0,11
074.3- Multiple Congenital Arthrogryposis	0,06	0,15	0,06	0,14	0,13
Q69.1- Supernumerary thumb(s)	0,06	0,07	0,13	0,13	0,11
Q71.6- Lobster claw hand	0,09	0,08	0,07	0,08	0,08
Q71.0- Complete congenital absence of the upper limb(s)	0,06	0,06	0,08	0,06	0,06
Q71.4- Radius longitudinal reduction defect | Club hand (congenital) | Radial hand	0,03	0,02	0,03	0,04	0,03
Q71.1- Congenital absence of the arm and forearm, with hand present	002	0,02	0,03	0,02	0,02
Q71.5- Longitudinal reduction defect of the ulna [ulna];	0,01	0,01	0,02	0,02	0,01

Regions of Brazil N- North; NE- northeast; CO- west center; SE- Southeast; S – South

Duplication defects, representing a group of CAUL, had the highest prevalence in all studied years, increasing from 3.6 cases per 10,000 LBs in 2010 to 4.8 in 2019.

Maternal and newborn variables were analyzed and are detailed in **Tables 4** and **5**. Concerning maternal age at the time of delivery, the majority of cases did not specify the age; however, children born to mothers over 40 years old exhibited a prevalence 1.36 times (or 36%) higher than those born to mothers under 40 years of age (OR = 1.36; 95% CI 1.19-1.56).

In terms of delivery type, Cesarean sections accounted for 12,418 cases of CAUL, with a prevalence of 7.6 per 10,000 LBs. In these cases, the prevalence of children born with CAUL was 1.07 times (or 7%) higher than in those born via spontaneous delivery (OR = 1.07; 95% CI 1.04-1.10). The number of prenatal visits was often unknown. Mothers who had three or fewer prenatal visits showed a 1.37 times (or 37%) higher prevalence of having children with CAUL compared to mothers who had four or more prenatal visits (OR = 1.37; 95% CI 1.27-1.48). Mothers with 11 years of education or less had a 1.22 times (or 22%) higher prevalence of having children with CAUL compared to those with 12 or more years of education (OR = 1.22; 95% CI 1.18-1.27).

Mothers with a gestational duration of 36 weeks or less were 1.89 times (or 89%) more likely to have children with CAUL than those with a gestational duration of 37 weeks or more (OR = 1.89; 95% CI 1.82-1.96). In cases of multiple pregnancies, such as twins or triplets, the prevalence of children born with CAUL was 1.29 times (or 29%) higher than in single pregnancies (OR = 1.37; 95% CI 1.27-1.48) (Table 4).

**Tabela 4:** Prevalence of CAUL according to maternal variables in Brazil for every 10,000 LB - 2010-2019

Variables		Prevalence	IC* 95%	
			Inferior limit	Upper limit
**Mother's Age**				
	20-34	2	2	2,13
	35-49	2	1,87	2,2
	40-44	2,7	2,4	3,18
	45-50	4,2	2,31	6,23
**Type of birth**				
	Cesarean section	7,6	7,55	7,82
	Vaginal	7,1	7,03	7,32
**Consultations/prenatal care**				
	1 to 3	3	2,77	3,27
	4 to 6	2,3	2,26	2,49
	≥ 7	1,9	1,88	2,01
**Mother's Education**				
	1 to 3 years	6,9	6,46	7,53
	4 to 7 years	7,5	7,34	7,8
	8 to 11 years old	7,9	7,77	8,04
	≥ 12 years	6,3	6,15	6,59
**Length of Pregnancy**				
	< 37 weeks	12,9	12,57	13,38
	37 to 41 weeks	6	6,77	6,98
	≥ to 42 weeks	6,9	6,38	7,56
**Type of Pregnancy**				
	Only	7,4	7,32	7,52
	Multiple	9,5	8,77	10,36

(*) CI – Confidence interval

When analyzing newborn variables related to birth weight, the highest prevalence of CAUL was observed in children weighing ≥ 2,499 g. Newborns in this weight range had a prevalence 2.64 times (or 64%) higher for CAUL compared to newborns with a birth weight ≥ 2,500 g (OR = 2.64; 95% CI 2.55-2.73).

Male newborns exhibited a 23% higher prevalence of CAUL than female newborns (OR = 1.23; 95% CI 1.17-1.30). Black newborns had an 88% higher prevalence of CAUL compared to newborns of other races (OR = 2.88; 95% CI 2.74-3.03). Regarding the Apgar score, the highest prevalence of CAUL was noted when the score was ≥ 7 at both the first (OR = 2.19; 95% CI 2.12-2.26) and fifth minutes (OR = 4.30; 95% CI 4.10-4.50) (Table 5).

**Tabela 5:** Prevalence of CAUL according to newborn variables in Brazil per 10,000 LB (2010-2019)

Variables		Prevalence	IC 95%	
			Inferior limit	Upper limit
**Birth weight**				
	≥ 2499 g	17,6	17,08	18,15
	2500 a 3999 g	6,5	6,42	6,62
	≥ 4000 g	6	6,2	7,05
**Sex**				
	Male	2,3	2,27	2,43
	Female	1,9	1,83	1,98
**Race**				
	White	6	6,66	6,97
	Black	12	12,18	13,37
	brown	7,5	7,37	7,56
	Yellow	6	4,84	7,9
	Indigenous	4	3,94	5,8
**Apgar 1st minute**				
	≥ 7	14	13,95	14,74
	> 7	6	6,47	6,67
**Apgar 5th minute**				
	≥ 7	30	28,76	31,43
	> 7	7	6,92	7,12

(*) IC- Confidence interval

**Figure 1:**
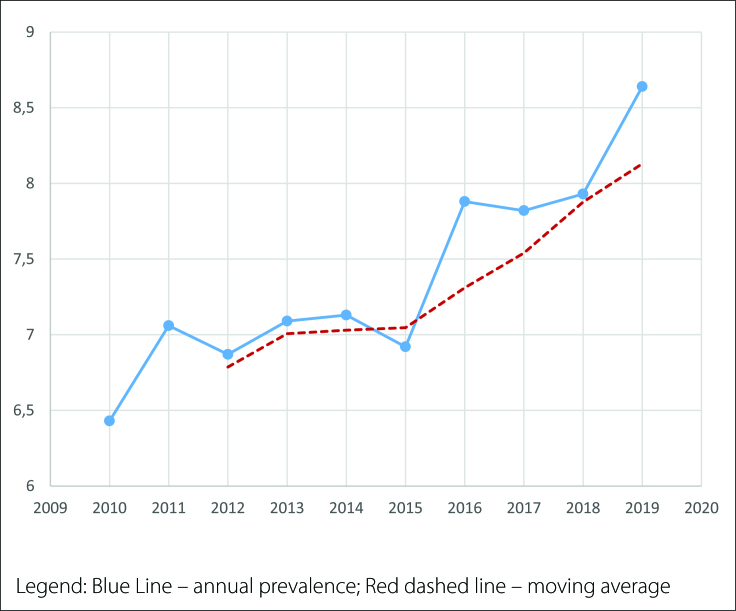
Linear trend based on the moving average – prevalence of CAUL for every 10,000 LB (2010-2019)

Source: DATASUS, SINASC 2010-2019

**Figure 2:**
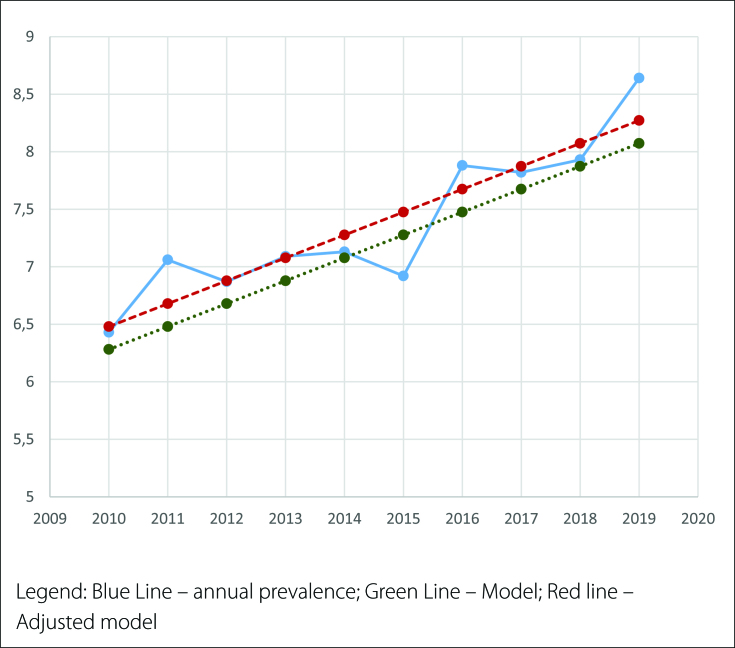
Linear trend - prevalence of CAUL for every 10,000 live births (2010-2019)

Source: DATASUS, SINASC 2010-2019

Through linear trend and moving average (MA) analysis of cases, an increase in prevalence was observed during the study period, with approximately 2.2 CAUL cases per 10,000 LBs when comparing 2010 and 2019. Linear regression analysis of the adjusted data for prevalence per 10,000 LBs showed an increase of 0.185 per year, with a standard error of 0.021. Thus, there was a linear trend of an increase in the prevalence of 0.206 (95% CI 0.133–0.237) CAUL per 10,000 LBs per year (P < 0.001). Notably, graph visualization demonstrated a linear trend component of increased CAUL prevalence when analyzing raw data over the years and smoothed moving averages (Figure 1). Remarkably, the graph visualization showed a linear trend component of increased CAUL prevalence when analyzing raw data over the years and the smoothed moving average (Figure 2).

## DISCUSSION

This study represents the first prevalence analysis of CAUL in Brazil, utilizing a national database to identify associated factors in newborns (NBs) and mothers. The national prevalence of upper limb anomalies between 2010 and 2019 was 7.5 per 10,000 LBs.

Several studies have examined the prevalence of CA in large populations. For instance, an assessment in New York, United States, based on state data, evaluated 4,883,072 children from 1992 to 2010, finding a CAUL prevalence of 27.2 cases per 10,000 births.^12^ Another study on the epidemiology of congenital limb anomalies in Japan estimated a prevalence of 4.15 per 10,000 LBs.^13^ In Finland, the national incidence of CAUL was observed at 5.25 per 10,000 LBs between 1993 and 2005, with these anomalies often associated with other congenital disabilities in up to two-thirds of cases.^14^

The data presented in this study are consistent with global findings on CAUL prevalence. In our analysis, the ICD code for supernumerary fingers (Q69.0) had the highest absolute number of cases with 11,708 and the highest prevalence at 4.02 cases per 10,000 LBs, comparable to findings in New York, where polydactyly was the most common CAUL, totaling 12,418 cases at a rate of 23.4 per 10,000 LB.^12^

Our study also analyzed maternal and newborn factors and their associations with CAUL. The findings indicate higher rates of CAUL in mothers over 40 years old, in preterm births (before 37 weeks), during multiple pregnancies, and among women who had fewer than seven prenatal visits.

In Tangará da Serra, Brazil, between 2006 and 2016, a study demonstrated a higher prevalence of CAs in newborns of mothers over 35 years old, an expected finding as maternal age is a primary risk factor for chromosomal anomalies.^15^

From 2010 to 2014, São Paulo reported 819,018 live births, 14,657 (1.6%) of which had CAs, predominantly osteoarticular and circulatory. An association was observed between congenital anomalies and maternal age over 40 years, multiple pregnancies, and newborns with low birth weight^16^, which aligns with our own results.

Examining factors associated with newborns, we found higher prevalence rates in those with low birth weight (< 2,500 g), male gender, Black race, and Apgar scores at both the 1st and 5th minute of ≥ 7. A study in Peru from 2009 to 2019 analyzed predictors of low Apgar scores and found that 65.3% of neonates with persistently low scores at 5 minutes had congenital anomalies, indicating a significant risk factor for low scores (OR = 5.81; P < 0.01). Notably, higher percentages of congenital anomalies were observed in newborns with birth weights < 1499 g (32.7% vs. 2.7%) and 1500–2499 g (11.9% vs. 7.2%) compared to controls, showing that birth weights < 1499 g (OR = 18.77; P < 0.01) and 1500–2499 g (OR = 2.51; P < 0.01) are significant risk factors for low Apgar scores.^25^

Between 2005 and 2014, 1,386,803 births occurred in Rio Grande do Sul, with diagnosed CA cases corresponding to an average overall rate of 9.2 per thousand. Higher rates of CAs were noted in mothers of newborns with Apgar scores less than 7, birth weights ≥ 1,500 g, and gestational ages ≥ 31 weeks. CAs were most frequently found in the intermuscular, nervous, and circulatory systems.^17^

In a study conducted in Rio de Janeiro between 1990 and 2002, the incidence of CAs in male newborns was higher, particularly in those born before 37 weeks with a birth weight of less than 2,500g^19^. Another study in Vale Paraíba Paulista identified a statistically significant association between gestational duration (< 37 weeks), lower Apgar scores (< 7), low birth weight (< 2,500 g), and CAs (P < 0.001).^18^

Several instruments are available for collecting epidemiological data to integrate and unify information on notifications of congenital anomalies. Established in 1974, the “International Clearinghouse for Birth Defects Surveillance and Research” (ICBDSR) aims to prevent congenital disabilities and currently includes 42 member programs worldwide.^21^ EUROCAT, a European network for epidemiological surveillance founded in 1979, now has 21 participating countries.^23^ This system has developed and matured over the past two decades through the standardization of definitions, diagnoses, and terminology.^24^

In Latin America, the Collaborative Latin American Study of Congenital Malformations, founded in 1967, covers South America, Costa Rica, and the Dominican Republic, employing a case-control methodology.^20^ However, a significant limitation in Brazil is the low participation of national maternity hospitals in this program, with only four of the 35 registered hospitals located in Brazil.^22^

We utilized data from DATASUS via the SINASC portal, a nationwide computerized data collection system where all birth-related data in Brazil are recorded. Given the country's vast size, this method offers rapid and convenient data collection and integration for public health, facilitating better analysis.

This study has limitations that should be considered when interpreting the results. Despite its nationwide scope and mandatory reporting, the SINASC database may contain inconsistencies, such as possible duplications, and does not allow for the individualization of cases, which would enable a more detailed statistical analysis of variables.

The cross-sectional nature of the study and the lack of individual case details regarding the exposure factor and disease at a specific time prevent establishing any cause-and-effect relationship between congenital anomalies and the analyzed variables.

The results underscore the significance of this research by providing a representative overview of the burden of CAUL among live births in Brazil. Multiple analyses facilitated an understanding of the variables associated with congenital anomalies. Enhancing the diagnosis of CAUL and ensuring the accurate completion of the Live Birth Certificate (DNV) through the ongoing education of health professionals responsible for record-keeping is a strategy that should be implemented by the Health Departments of Brazilian states to minimize the incidence of missing or inaccurate data, thereby reducing underreporting.

This nationwide study was conducted in a country with a continental span. Nearly 30 million cases over ten years were analyzed. A national computerized reporting system that allows for the rapid and precise exchange of information across distant states and municipalities is invaluable.

## CONCLUSION

The prevalence of CAUL in Brazil between 2010 and 2019 was 7.5 per 10,000 LBs. ICD Q69.0, representing supernumerary fingers, is the most common CAUL in our population. The maternal factors associated with CAUL included being under 40 years of age, undergoing cesarean delivery, having fewer than three prenatal consultations, having less than 11 years of education, a gestational age of 36 weeks or less, and experiencing multiple pregnancies. For newborns, associated factors included a birth weight of 2,500 grams or less, male gender, Black race, and Apgar scores of 7 or less at both the 1st and 5th minutes.

A consistent upward trend in CAUL case reports has been observed over the past decade. This study can inform more effective public health policy strategies. However, further research is essential to enhance our understanding of the underlying causes of the increase in CAUL cases, particularly concerning supernumerary fingers and their implications.
